# Global warming changes tropical cyclone translation speed

**DOI:** 10.1038/s41467-019-13902-y

**Published:** 2020-01-08

**Authors:** Munehiko Yamaguchi, Johnny C. L. Chan, Il-Ju Moon, Kohei Yoshida, Ryo Mizuta

**Affiliations:** 1 Meteorological Research Institute, Japan Meteorological Agency, Tsukuba, Ibaraki Japan; 2 Guy Carpenter Asia-Pacific Climate Impact Centre, School of Energy and Environment, City University of Hong Kong, Kowloon, Hong Kong China; 3 Typhoon Research Center, Jeju National University, Jeju, South Korea

**Keywords:** Atmospheric science, Climate change, Climate and Earth system modelling, Projection and prediction

## Abstract

Slow-moving tropical cyclones (TCs) can cause heavy rain because of their duration of influence. Combined with expected increase in rain rates associated with TCs in a warmer climate, there is growing interest in TC translation speed in the past and future. Here we present that a slowdown trend of the translation speed is not simulated for the period 1951–2011 based on historical model simulations. We also find that the annual-mean translation speed could increase under global warming. Although previous studies show large uncertainties in the future projections of TC characteristics, our model simulations show that the average TC translation speed at higher latitudes becomes smaller in a warmer climate, but the relative frequency of TCs at higher latitudes increases. Since the translation speed is much larger in the extratropics, the increase in the relative frequency of TCs at higher latitudes compensates the reduction of the translation speed there, leading to a global mean increase in TC translation speed.

## Abstract (web summary)

How the translation speed of tropical cyclones has changed in recent decades and will change in the future has been the subject of debate. Model results show that on average, they have not slowed down in the past, but despite a slowing of tropical cyclones at higher latitudes, a poleward shift in their mean track location causes a general speed up under high greenhouse gas emissions.

## Introduction

Tropical cyclones (TCs) are one of the most intense weather systems in the world and thus it is of great importance to better understand how the behavior of TCs may change under global warming such as their frequency and intensity. Translation speed of TCs is one important feature because the slower TCs move, the longer is their influence time, and the greater is the impact of severe weather events associated with TCs such as heavy rain and strong winds^1,2^. In relation to the anticipated increase in precipitation associated with TCs in the future^3,4^, how the translation speed of TCs has changed in recent decades and will change in a future warmer climate is an area of active research^5–8^. Kossin^5,6^ (hereafter, K18) concluded, based on observational data (known as best-track data; see Data availability), that the translation speed of TCs has decreased globally by 10% between 1949 and 2016, possibly due to global warming. The magnitude of the slowdown varies from basin to basin, with the largest slowdown being in the western North Pacific basin. However, Moon et al.^7^ (hereafter, M19) cast doubts on K18’s conclusions, pointing out that such a slowdown trend arose mainly due to the lack of detection of weak and over-the-sea TCs, which have a relatively slow translation speed, in the pre-satellite era (1965 and before). Because it is impossible to reproduce the observational data during the pre-satellite era with the same quality as those in the post-satellite era, we propose to analyze results of numerical simulations for the current and future climates.

Here the following two questions are at the center of discussion in this study. The first question is whether there exists a decreasing trend in the translation speed of TCs during the historical period, and the second is how the translation speed might change in the future warmer climate. Results from the model used in this study show that the global mean TC translation speed has not slowed down in the past, but under global warming, they will in general speed up due to a poleward shift in their mean track location, although those moving into the extratropics slow down.

## Results

### Reproducibility of observed translation speeds by model simulations

**Fig. 1:**
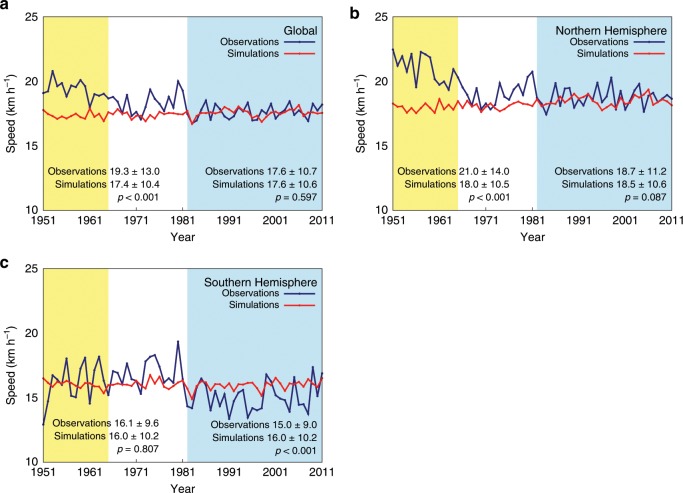
Global and hemispheric time series of annual-mean tropical cyclone translation speed from the observations and simulations. **a**–**c** The period of the time series is 1951–2011. Navy blue (red) line is for the observations (simulations). Yellow (blue) shading indicates the pre-satellite (post-geostationary satellite) era. Time series are shown for the global (**a**) data and for the Northern (**b**) and Southern (**c**) Hemisphere data. The period average annual-mean translation speed and one standard deviation in the pre-satellite (post-geostationary satellite) era are shown with *p* value using the two-tailed Student’s *t* test to assess the statistical significance of the difference between the observations and simulations in the bottom left (right) corner of each plot.

**Fig. 2:**
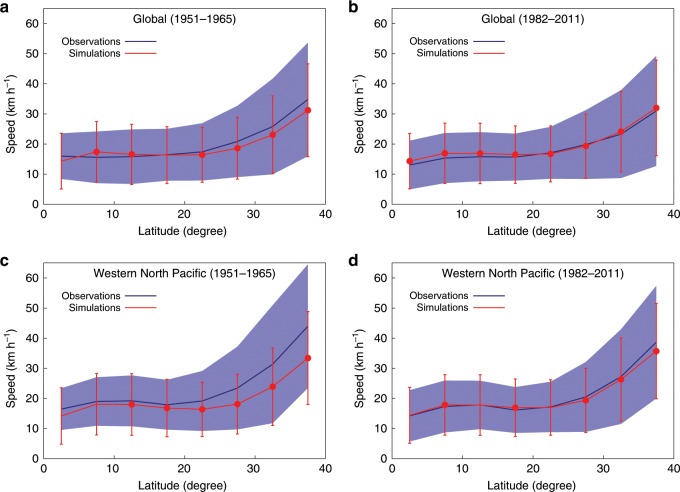
Global and Western North Pacific basin mean tropical cyclone translation speed against latitudes from the observations and simulations **a**–**d** The range of the latitude is 0–40° with 5° interval. Navy blue (red) line is for the observations (simulations). Vertical shading (bars) indicates 1 standard deviation in the bins for the observations (simulations). The latitudinal distribution is shown for the global data over the pre-satellite era (**a**) and over the post-geostationary satellite era (**b**), and for the Western North Pacific basin data over the pre-satellite era (**c**) and over the post-geostationary satellite era (**d**). Red dots mean the difference between the observations and simulations is statistically significant at a 99% level (*p* < 0.01, two-tailed Student’s *t* test).

In this study, we use results of high-resolution large-ensemble simulations^9,10^ (see Methods). It is important that these simulations reproduce important aspects of the observed TC frequency and distribution under current climate conditions in order to address the above questions with some confidence. Yoshida et al.^10^ (hereafter, Y17) showed that the simulated annual TC genesis and TC distribution match the observations quite well (see Figs. 1 and 2a, c of Y17). Figure 1 shows the time series of annual-mean translation speed of TCs from the observations and simulations for the current climate for the globe and the Northern and Southern Hemispheres, respectively (see Methods). Here the primary period of comparison between the observations and simulations is limited to 1982 to 2011 when data from geostationary satellites are available globally. It is relatively unlikely that any TC would go undetected in the post-geostationary satellite era, as compared to the pre-satellite era, and thus the best-track data are almost certainly more homogeneous and reliable since 1982. The simulations reproduce the observation-based global annual-mean translation speed in the post-geostationary satellite era very well, with both the observations and simulations having a period average of 17.6 km/h. The degree of similarity between the observations and simulations varies by the hemisphere and basin, but it is relatively high in the western North Pacific, North Atlantic, Northern Indian, and South Pacific basins (Supplementary Fig. 1).

Another piece of evidence that the simulations reproduce the TC translation speed of the current climate and can be used to assess it is the reproducibility of their latitudinal distribution. Figure 2 shows the TC translation speed against latitudes globally and in the western North Pacific basin, respectively. In the evaluation for the post-geostationary satellite era (Fig. 2b, d), the simulations reproduce the latitudinal distribution of translation speed relatively well.

There are, however, some deficiencies in the simulations. For example, differences in the translation speed between the observations and simulations can be seen in some basins such as the eastern North Pacific basin even in the post-geostationary satellite era (Supplementary Fig. 1). The standard deviation of the translation speed in the simulations is smaller than that of the observations in the North Atlantic basin (note that smaller year-to-year variations in the annual-mean translation speed in the simulations is due to the smoothing resulting from averaging a large number of ensemble simulations). But overall it can be concluded that the simulations reproduce the translation speed of the observations in the post-satellite era realistically and can therefore be used to compare with observations for trend analyses.

### TC translation speed in the past

Returning to the first question as to whether there exists a slowdown trend in the translation speed of TCs in the current climate, neither global nor hemispheric model simulation results show a decrease in the translation speed from 1951 to 2011 (Fig. 1). This is also true for simulations in each TC basin (Supplementary Fig. 1). Another point to notice from Fig. 1 is that the translation speed based on the observations is larger than that from the simulations in the pre-satellite era, and the difference between them is notably seen globally and in the western North Pacific basin. Such discrepancy between the observations and simulations is also clearly seen in the comparison of the latitudinal distributions of the translation speed (Fig. 2a, c). These results suggest that, consistent with the conclusions of M19, inhomogeneities in the observational data may be one of the reasons for the decrease in the translation speed proposed by K18.

### TC translation speed in the future

The high-resolution large-ensemble simulations were conducted not only for the current climate but also for the future climate over the period 2051–2110 assuming a 4 K surface warming from the pre-industrial level (see Methods). Comparisons of the simulation results between the current and future climates enable us to explore the second question: how the translation speed might change in a future warmer climate. Table 1 shows the comparison of the annual-mean TC translation speed for the current and future climates. The translation speed averaged over 1951–2011 and over 2051–2110 globally is 17.5 and 18.0 km/h, respectively, suggesting that the translation speed may increase in the future. A statistically significant increase is also simulated for some individual basins, including the western North Pacific, North Atlantic, and South Pacific basins.

**Table 1:** Changes in the tropical cyclone translation speed between the current and future climates.

	Current climate	Future Climate	*p* Value
Global	17.5	18.0	<0.001
Northern Hemisphere	18.3	18.6	<0.001
Southern Hemisphere	16.0	16.3	<0.001
North Atlantic	22.1	22.6	<0.001
Western North Pacific	18.2	18.4	0.017
Eastern North Pacific	18.5	18.7	0.305
Northern Indian	13.8	13.7	0.106
Southern Indian	15.9	15.8	0.140
South Pacific	16.1	17.0	<0.001

The translation speeds (km/h) are either a global, hemispheric, or basin mean. The periods of the current and future climate are 1951–2011 and 2051–2110, respectively. The *p* values using the two-tailed Student’s *t* test show the statistical significance of the difference between the current and future climates

**Fig. 3:**
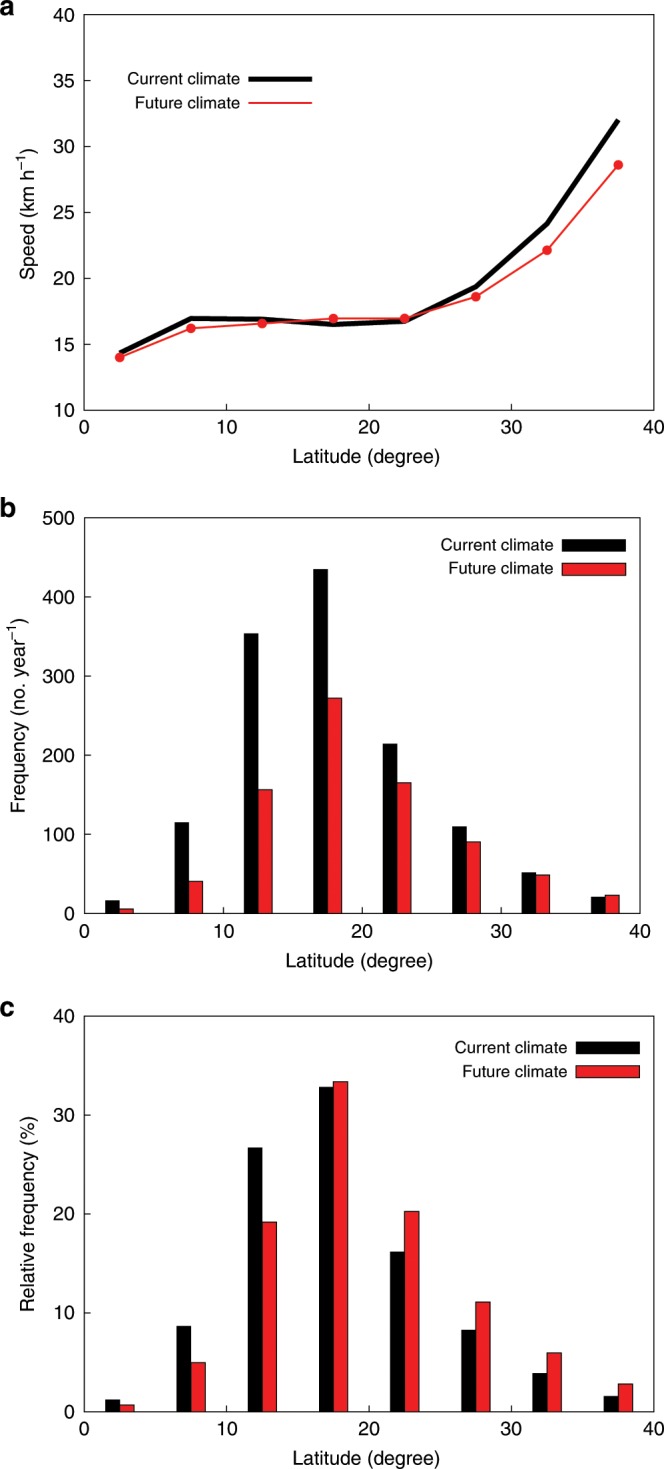
Changes in the tropical cyclone translation speed, tropical cyclone absolute, and relative frequency against latitudes. **a**–**c** The range of the latitude is 0–40° with 5° interval, and the speed and both frequencies are globally averaged at each latitudinal bin. Red (black) line (**a**) and boxes (**b**, **c**) is for the future (current) climate. The difference between the current and future climates is statistically significant at a 99% level (*p* < 0.01, two-tailed Student’s *t* test) at each bin for **a**–**c**.

Care must be taken in interpreting this result. In a future warmer climate, the westerly winds are expected to weaken due to the decrease in baroclinicity in the atmosphere. Since the westerly winds play an important role as the steering flow of TCs, these weakened westerly winds will lead to a reduction in the TC translation speed in the extratropics (Supplementary Fig. 2). Figure 3a shows the latitudinal distribution of the translation speed in the current and future climates in the global domain. In a warmer climate, the average translation speed decreases in the extratropics compared to the current climate. A similar translation speed decrease in the extratropics is seen in each TC basin except for the Southern Indian Ocean basin (Supplementary Fig. 3).

Figure 3b, c (Supplementary Figs. 4 and 5) show changes in the absolute and relative frequency of TCs over the globe (each TC basin), respectively (see Methods). A relatively large reduction in the TC absolute frequency in the tropics and sub-tropics results in an increase in the relative frequency of TCs in the extratropics. This result holds even when the samples are limited to categories 4 and 5 TCs (Supplementary Fig. 6). Because the TC translation speed in the extratropics is much larger than that in the tropics and sub-tropics, the increase in the relative frequency of TCs in the extratropics could result in an increase in the global annual-mean translation speed in spite of the slowdown of the translation speed in the extratropics. In other words, the annual global mean translation speed change in a warmer climate depends on which is the dominant contribution: the reduction of the average translation speed in the extratropics or the poleward shift of TC frequency.

## Discussion

The increase in relative frequency of TCs in the extratropics seen in the model simulations used in this study can be inferred from other model simulation studies through geographical distribution of TC frequency difference between the current and future warmer climates^11,12^. The decrease in TC frequency at around 15° North/South in the North Atlantic, western North Pacific, and the Southern Hemisphere in a warmer climate, which leads to the increase in relative frequency of TCs in the extratropics, is a common feature among the model simulations. Wehner et al.^13^ directly assessed the future change in relative frequency of TCs with latitudes. Their results are qualitatively the same as those from our model simulations: decrease at around 15° North/South or less and an increase at around 20° North/South or more.

It is also true that there are large uncertainties in the future projections of various characteristics of TC activity^14,15^. For example, some model simulations suggest an increase in the global^16,17^ and western North Pacific^18,19^ TC frequency under a warmer climate. Nakamura et al.^20^ showed through multi-model analyses that there is a large range of uncertainty across numerical simulations as to whether a poleward shift of TC occurrence exists in the western North Pacific basin, where there has been the largest trend in the observations for the lifetime maximum intensity of TCs^21^ and TC genesis location^22^, although the combined multi-model signal has a statistically significant poleward shift in a warmer climate. In the North Atlantic, Daloz et al.^23^ did not find any significant TC track changes in the future. Ramsay et al.^24^ did not find a consistent poleward shift in the Southern Hemisphere TC tracks across multiple models, either. These previous studies could potentially lead to a different conclusion on the future change in the TC translation speed from this study. The bimodal distribution of TC intensity proposed by Lee et al.^25^ may not be represented well by our 60-km atmospheric model, which could potentially modify our conclusions. In addition, an atmosphere–ocean coupling effect could have a role in modulating the relative frequency of TCs. For example, some intense TCs could reach higher latitudes without influence of ocean cooling, while an ocean subsurface warming might cause an increase in the TC frequency^26,27^. Thus further investigation using an ocean-coupled model with finer grid spacing will be needed in the future.

**Fig. 4:**
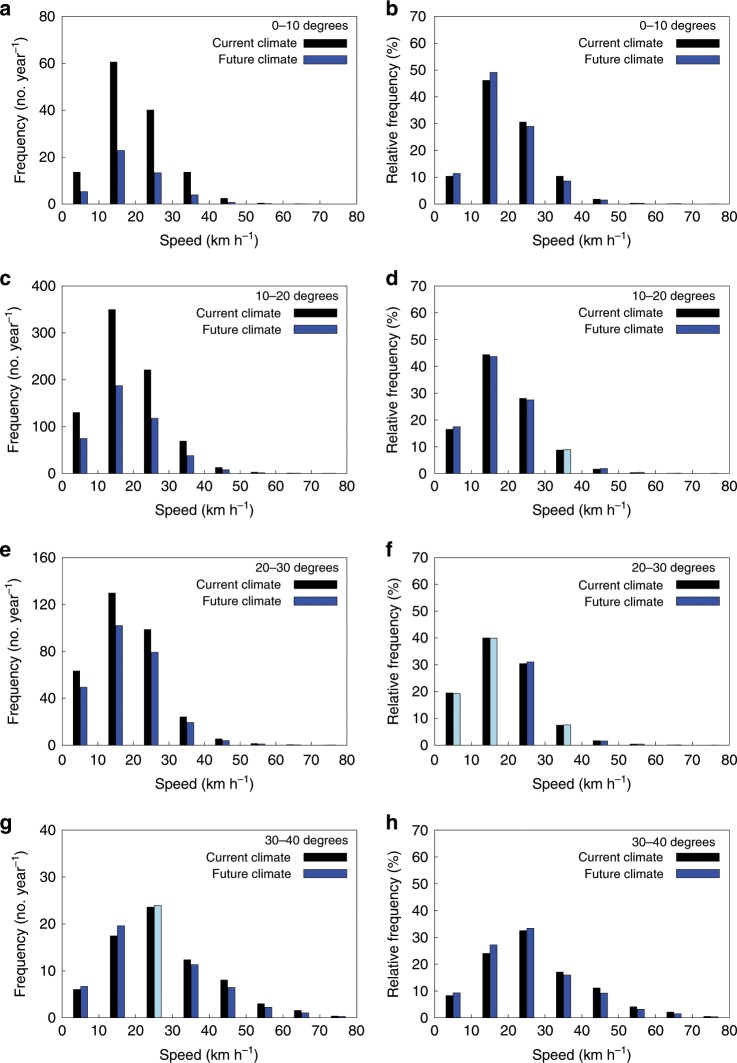
Changes in the tropical cyclone absolute and relative frequency against translation speeds. **a**–**h** The range of the translation speed is 0–80 (km/h) with 10 (km/h) interval, and absolute (**a**, **c**, **e**, **g**) and relative (**b**, **d**, **f**, **h**) frequencies are globally averaged at each translation speed bin and at each latitudinal band from 0° to 10° (**a**, **b**), 10° to 20° (**c**, **d**), 20° to 30° (**e**, **f**), and 30° to 40° (**g**, **h**). Blue and cyan boxes are for the future climate and black boxes are for the current climate. Blue (cyan) means that the difference between the current and future climates is (not) statistically significant at a 99% level (*p* < 0.01, two-tailed Student’s *t* test).

The global or basin mean translation speed of TCs really depends on the latitudinal distribution of TCs. The mean translation speed alone can be misleading when discussing the future change in the translation speed at a certain latitude and assessing its social and economic impact. Thus, the whole histogram of TC translation speed at each latitudinal band is also an important metric. Figure 4 shows the histogram of the TC translation speed at each latitudinal band with 10° interval. The relative frequency of TC translation speed of 20 km/h or less increases in the future at a 0–10° latitude band, but it does not mean that the absolute number of such slow-moving TCs increases there in the future compared to the current climate. The difference between the current and future climates is more clearly seen at a 30–40° latitude band. Both the absolute and relative frequencies of TC translation speed of 30 km/h or less (more) tend to increase (decrease) in the future, although there is a large variability among the basins (Supplementary Fig. 7).

There are two important conclusions from the results of this study that are significant for both the scientific and disaster-preparedness communities. First, historical simulations for 1951–2011 suggest that there has been no significant decrease in the translation speed of TCs over that period, which is consistent with the arguments of M19. Second, with an increase in the relative frequency of TCs at higher latitudes in a warmer future climate, it is expected that the global average translation speed could be higher than that in the present day.

Care must be taken in the interpretation of this second conclusion. The mean translation speed metric has been used in this study for comparison with K18 and M19. However, it highly depends on the frequency of TC occurrence at various latitudes and in different basins and would not be a meaningful metric for disaster risk prevention and for assessing the potential impact of TCs associated with their influence of time at a given location. Thus, an important implication of this study for the disaster-preparedness communities, especially for those at higher latitudes, is that while it is likely that TC translation speeds in the tropics would remain largely unchanged under global warming, those at the higher latitudes would decrease in spite of the fact that the global and/or basin-wide mean translation speeds may increase in the future. Furthermore, the poleward shift of the relative frequency of TCs is fundamental for the conclusions of this study, but previous studies show that there are large uncertainties in projections of changes in TC tracks simulated in numerical models. Thus, further investigation will be needed in the future to better understand the causes of such uncertainties as well as to reduce them.

## Methods

### Model descriptions

The large-ensemble simulations were conducted by an atmospheric global circulation model (AGCM). The AGCM used in this study is the Meteorological Research Institute AGCM, version 3.2 (MRI-AGCM3.2) with a 60 km horizontal grid spacing, which is exactly the same as MRI-AGCM3.2H listed in the CMIP5 archive^28^. In the simulations for the historical period, the observed monthly mean sea surface temperature (SST) and sea ice concentration from COBE-SST2^29^ are used as the lower boundary conditions. The period of the simulations is 60 years (1951–2010) and the ensemble size is 100. In addition to using different initial conditions, small perturbations of SST based on its analysis error are adopted for the ensemble experiments. Global mean concentrations of greenhouse gases are set to the observational values for each year. A future climate scenario in which the global mean surface air temperature is assumed to be 4 K warmer than the pre-industrial climate is simulated, corresponding to the global warming around the end of the twenty-first century under the representative concentration pathway 8.5 (RCP8.5) scenario of CMIP5. The amplitude of the warming is kept constant throughout the simulations. The period of the simulations is 60 years and the ensemble size is 90. Six CMIP5 models are used for obtaining the warming pattern of the SST and each pattern is added to the observational SST of 1951–2010. For each warming pattern, 15-member ensemble experiments are conducted using different initial conditions and sea surface perturbations for each SST change.

### TC tracking method

The TC tracking is based on the model outputs, including sea level pressure, 850 hPa relative vorticity, 850 hPa and surface wind speed, vertical wind shear between 850 and 300 hPa, warm core temperature, and duration period^10,28^. Appropriate threshold values of these variables are chosen so that the global number of TC geneses detected in the current climate simulations by MRI-AGCM3.2H is close to that observed. Compared to other explicit detection methods, the TC tracking method in the present study does not show large differences in TC detection^30^.

### Calculation of translation speed and frequency

The TC tracking data include the central position of TCs every 6 h. For each ensemble member and each year, we calculated great-circle distance between two locations along TC tracks and computed the annual average translation speed of the ensemble member for the year. We repeated this computation for all ensemble members and then took an ensemble mean to obtain the annual-mean translation speed. The absolute frequency is calculated by counting the number of 6-hourly tracked TC central locations that meet the conditions of the latitudinal or translation speed bins.

## Supplementary information

Supplementary Information

## Data Availability

The best-track data were taken from the International Best Track Archive for Climate Stewardship (IBTrACS; https://www.ncdc.noaa.gov/ibtracs/). The tropical cyclone tracking data from the simulations are available from the corresponding author on request.

## Code availability

All codes used to analyze and plot the data are available from the corresponding author on request.
