# Supplementary material for: Global warming changes tropical cyclone translation speed
Source: Nat Commun. 2020 Jan 8;11:47. doi: 10.1038/s41467-019-13902-y (PMC6949250; doi:10.1038/s41467-019-13902-y)
Supplement: Supplementary file 1 — Supplementary Information [file 41467_2019_13902_MOESM1_ESM.pdf]

Supplementary Information

Global Warming Changes Tropical Cyclone Translation Speed

Yamaguchi et al.

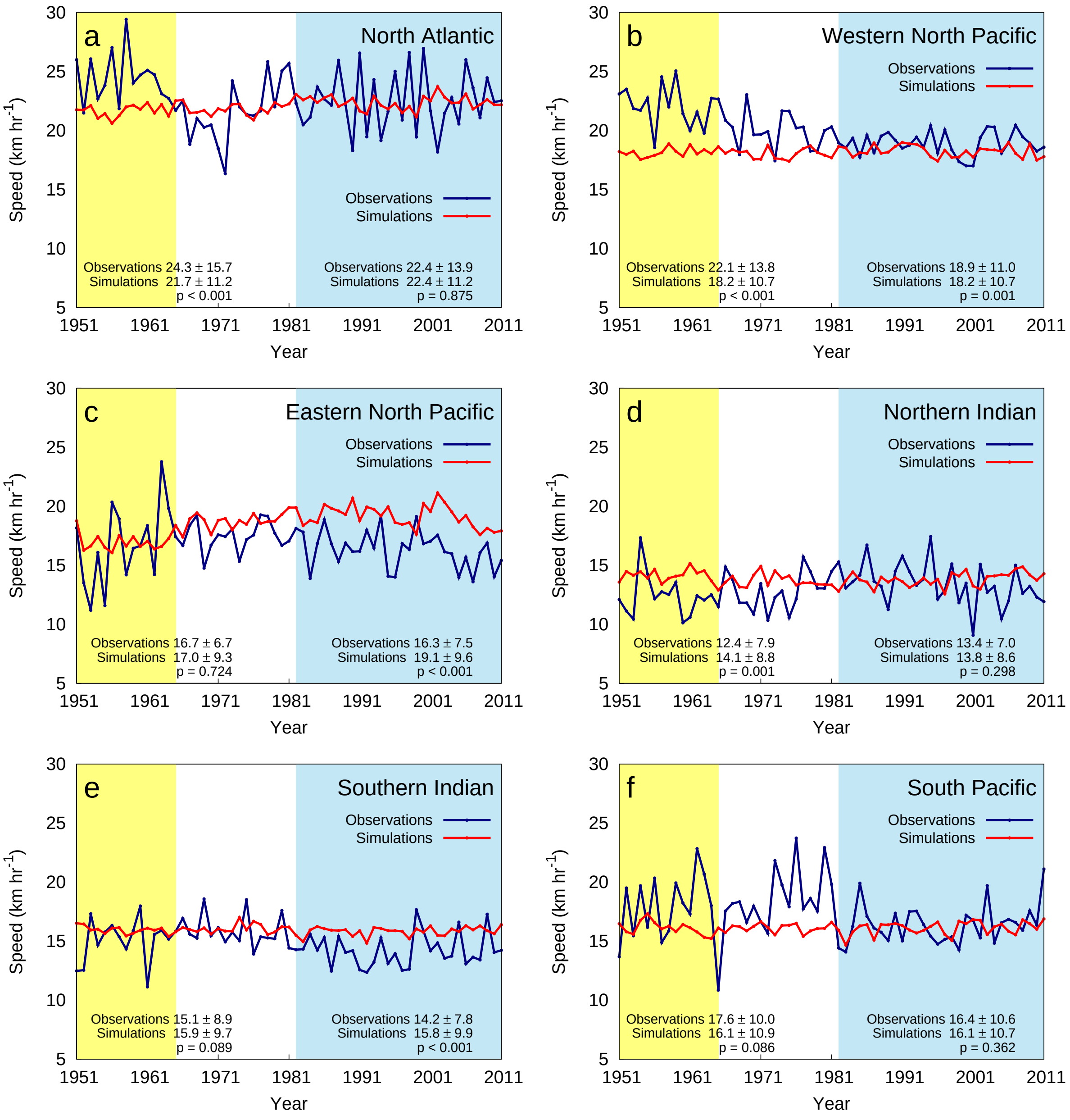

Supplementary Figure 1: Time series of annual-mean tropical cyclone translation speed from the observations and simulations at each tropical cyclone basin. a-f, The period of the time series is 1951-2011. Navy blue (red) line is for the observations (simulations). Yellow (blue) shading indicates the pre-satellite (post-geostationary satellite) era. Time series are shown for the North Atlantic (a), Western North Pacific (b), Eastern North Pacific (c), Northern Indian (d), Southern Indian (e), and South Pacific (f) basins, respectively.

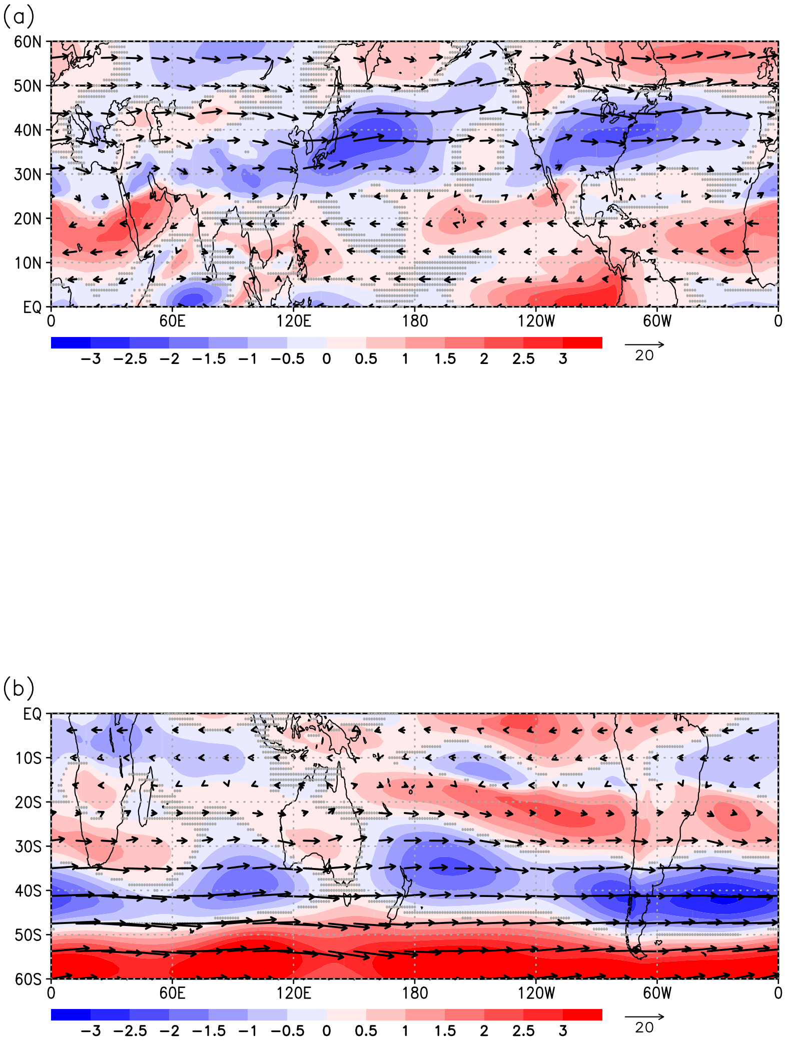

Supplementary Figure 2: Changes in the atmospheric environments between the current and future climates. a,b, Vectors indicate 4-month average 500 hPa winds in the current climate and shades indicate changes in the wind speeds in the future. The unit is m s^{-1}. The wind vectors and the wind speed changes are shown for the Northern Hemisphere (a) and Southern Hemisphere (b) as 4-month average over July-October and January-April, respectively. Dots indicate regions where the difference of the wind speeds between the current and future climates are not statistically significant at a 99 % level.

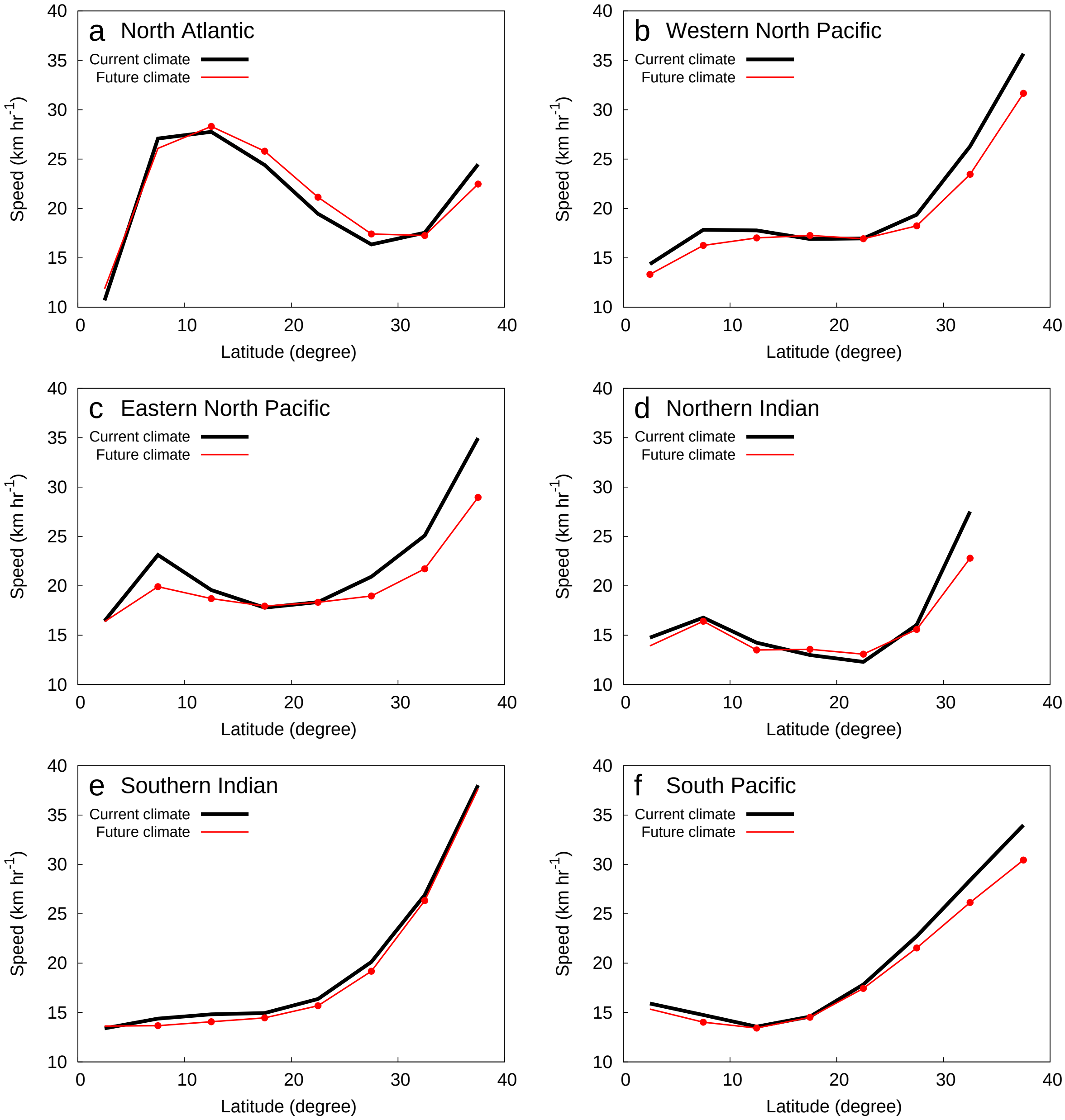

Supplementary Figure 3: Changes in the tropical cyclone translation speed at each tropical cyclone basin against latitudes. a-f, The range of the latitude is 0-40 degrees with 5 degrees interval, and the speed is averaged at each latitudinal bin over the North Atlantic (a), Western North Pacific (b), Eastern North Pacific (c), Northern Indian (d), Southern Indian (e), and South Pacific (f) basins, respectively. Red (black) line is for the future (current) climate. Red dots mean the difference between the current and future climates is statistically significant at a 99 % level ($p < 0.01$, two-tailed Student's t-test).

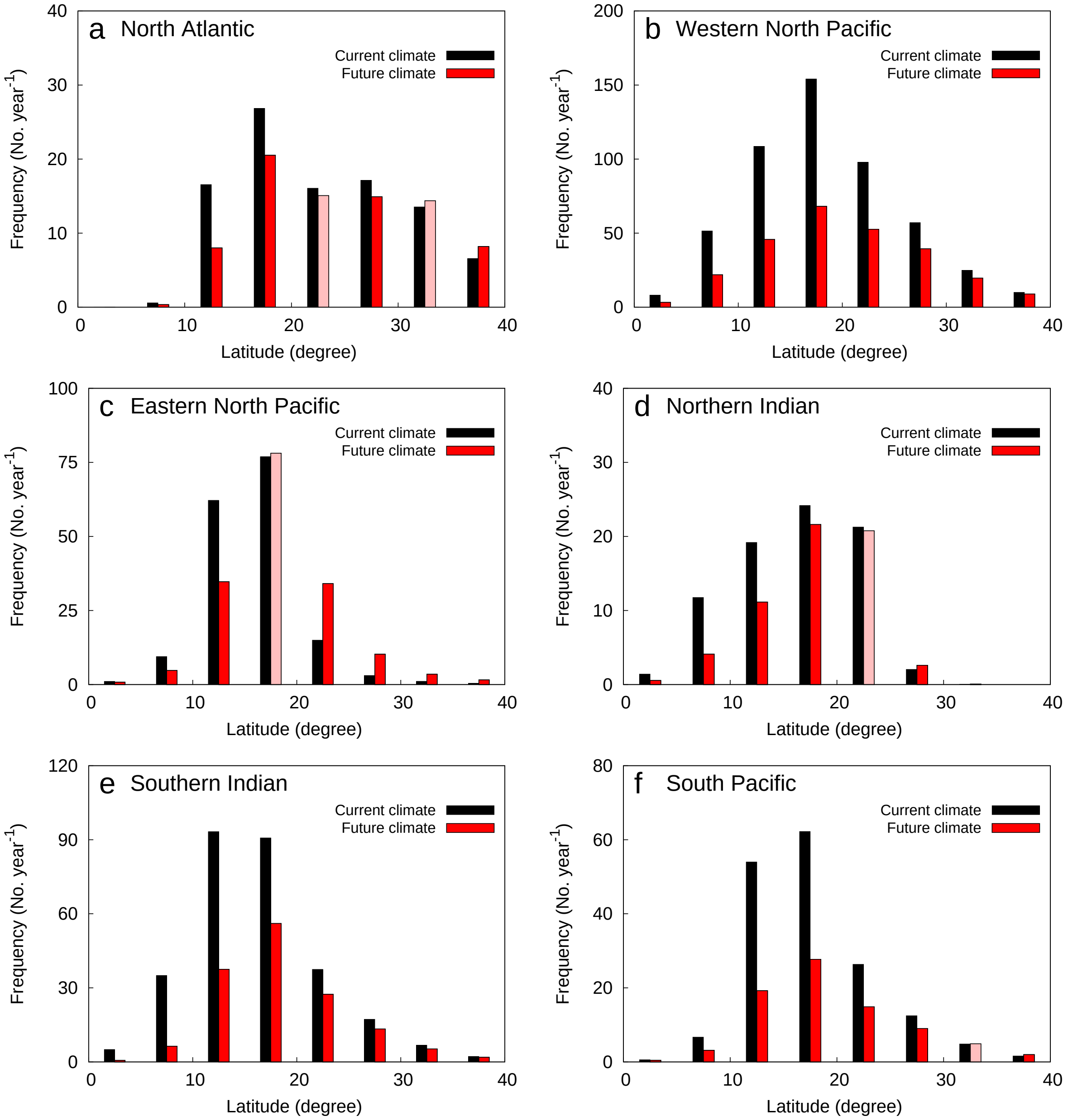

Supplementary Figure 4: Changes in the tropical cyclone absolute frequency at each tropical cyclone basin against latitudes. a-f, The range of the latitude is 0-40 degrees with 5 degrees interval, and the absolute frequency is averaged at each latitudinal bin over the North Atlantic (a), Western North Pacific (b), Eastern North Pacific (c), Northern Indian (d), Southern Indian (e), and South Pacific (f) basins, respectively. Red/pink (black) boxes are for the future (current) climate. Red (pink) boxes mean the difference between the current and future climates is (not) statistically significant at a 99 % level ($p < 0.01$, two-tailed Student's t-test).

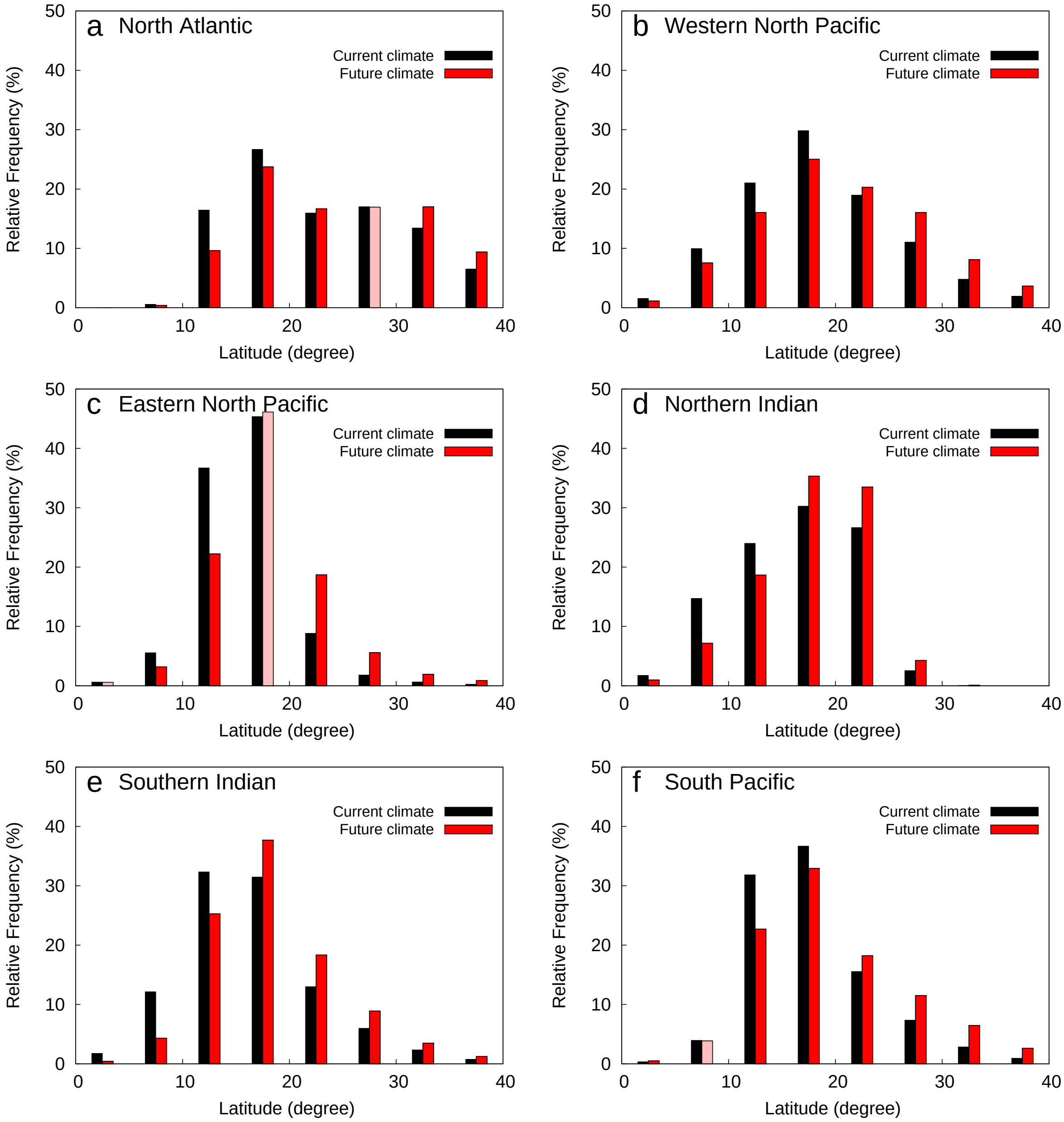

Supplementary Figure 5: Changes in the tropical cyclone relative frequency at each tropical cyclone basin against latitudes. a-f, The range of the latitude is 0-40 degrees with 5 degrees interval, and the relative frequency is averaged at each latitudinal bin over the North Atlantic (a), Western North Pacific (b), Eastern North Pacific (c), Northern Indian (d), Southern Indian (e), and South Pacific (f) basins, respectively. Red/pink (black) boxes are for the future (current) climate. Red (pink) boxes mean the difference between the current and future climates is (not) statistically significant at a 99 % level ($p < 0.01$, two-tailed Student's t-test).

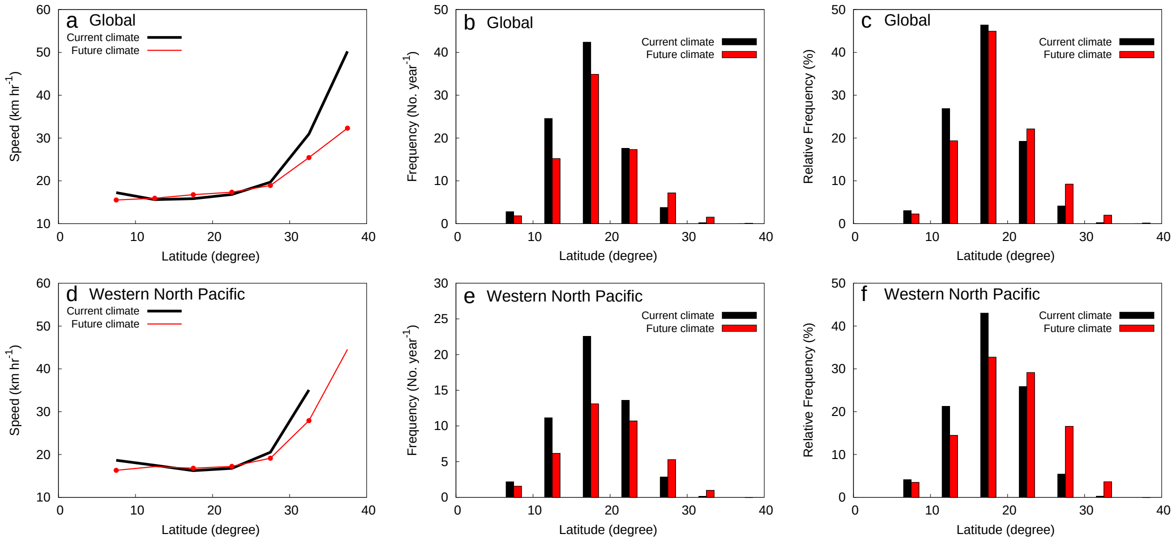

Supplementary Figure 6: Changes in the tropical cyclone translation speed, tropical cyclone absolute and relative frequency against latitudes for categories 4 and 5 tropical cyclones. a-f, The range of the latitude is 0-40 degrees with 5 degrees interval, and the speed and both frequencies are averaged at each latitudinal bin over the globe (a-c) and the Western North Pacific basin (d-f), respectively. Red (black) line (a,d) and boxes (b,c,e,f) are for the future (current) climate. The difference between the current and future climates is statistically significant at a 99 % level ($p < 0.01$, two-tailed Student's t-test) at each bin for a-f.

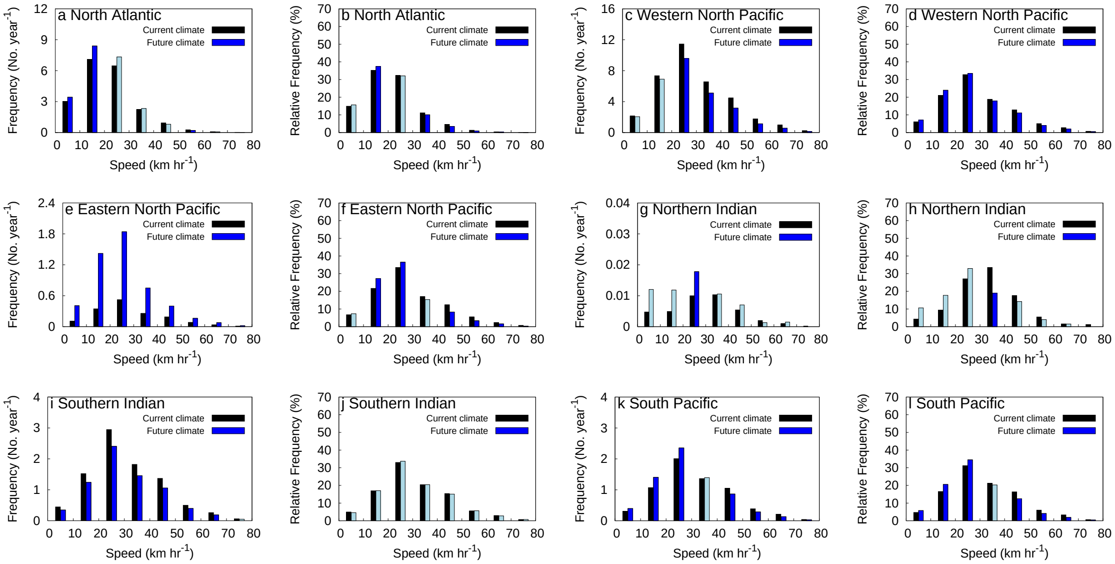

Supplementary Figure 7: Changes in the tropical cyclone absolute and relative frequency against translation speeds at each tropical cyclone basin. a-l, The range of the translation speed is 0-80 km hour^{-1} with 10 km hour^{-1} interval, and absolute (a,c,e,g,i,k) and relative (b,d,f,h,j,l) frequencies are averaged at each translation speed bin at a latitudinal band of 30-40 degrees over the North Atlantic (a,b), Western North Pacific (c,d), Eastern North Pacific (e,f), Northern Indian (g,h), Southern Indian (i,j), and South Pacific (k,l) basins, respectively. Blue/cyan (black) boxes are for the future (current) climate. Blue (cyan) means that the difference between the current and future climates is (not) statistically significant at a 99 % level ($p < 0.01$, two-tailed Student's t-test).
